# The Treatment of Scarlet Fever

**Published:** 1908-02

**Authors:** A. Gy, M. Claret

**Affiliations:** Paris; Paris


### CLINICAL LECTURE

### The Treatment of Scarlet Fever[1]

By DR. A. GY and DR. M. CLARET, Paris.

[*La Tribune Medicale, December, 1907.*]

The prognosis of scarlet fever so far as life is concerned is favorable, for various statistics give a mortality of about 1.5 per cent., yet the frequency of special complications and the sequelæ of these complications as well as the possibility of preventing them require much thought on the part of the physician as regards the treatment of this disease. If this were pursued with proper understanding there would be fewer cases of nephritis developing at an adult age and leading often to a fatal end as the result of the involvement of the kidney during scarlet fever in childhood in which nothing but simple rest in bed had been employed.

Although we do not know as yet the exact infectious agent of scarlatina we know that the disease is of microbic origin. Many attempts have been made to obtain a serum against this disease but so far very little that is conclusive has been obtained.

There are three classes of serums employed in scarlet fever at present. Some authors, struck by the almost constant presence of streptococci in the exudates of scarlet fever patients regard these germs as the specific agent of the disease and employ the serum of animals rendered immune against the streptococci derived from various sources. Marmoreck obtained good results with a serum and similar experiences were reported by Baginsky, Marfan, Comby, and others. The serum prepared by Moser, which also was prominently discussed some time ago, has practically been forgotten today. Another group of observers felt uncertain as to the specific microbe but were sure that a first attack of scarlet fever rendered the patient immune and therefore looked for specific antitoxins in the blood of patients who had recovered from the disease. Roger and Leyden injected into scarlet fever patients some of the serum of other patients who had recovered from the disease and reported some favorable cases, but this treatment had to be abandoned on account of the difficulty of obtaining the serum in sufficient quantities.

Kampe's serum has been prepared from the epidermal scales of patients. These we know to contain the contagion of scarlet fever, whatever that may be. Kampe immunised animals with the poisonous products extracted from these scales and found that the serum of these animals had both preventive and curative action. The good results which he reports in 200 cases may be attributed to a benign series of cases. Two years after he introduced his serum it was forgotten.

In brief, the specific treatment of scarlet fever is still to be found, so that we are obliged to rely upon hygiene and therapeutics for the proper means of securing as benign a course as possible of this disease. This brings us to the consideration of the subject of this lecture.

In the normal course of scarlatina without complications the treatment will be chiefly hygienic and directed toward the prevention of complications. In the first part of our discourse we will consider the hygiene of scarlet fever which, as we shall see, is of great importance. In a second chapter we shall consider the various complications and their treatment.

*Hygiene of Scarlatina.*—When we find a child presenting all the typical symptoms of scarlet fever yet without any tendency to complications, in other words, a scarlatina tending toward a normal favorable course, three points should occupy our attention: (1) the general hygiene of the room of the patient; (2) the general and local antisepsis intended to prevent the development of pyogenic germs which give rise to complications; (3) the diet of the patient with special reference to the prevention of scarlatinal nephritis. Several problems will also arise concerning the prophylaxis of the case and the prevention of the spread of the disease.

*Hygiene of the Room.*—It is customary in the medical textbooks, in speaking of the treatment of all the eruptive fevers, to recommend good ventilation and the maintenance of a constant temperature about the bed of the patient, for those physicians who have seen their private patients shut up in rooms that are never opened and covered with innumerable quilts for the purpose of "making the eruption come out" or of "preventing it from going inside," some details will not seem useless and their application will require much diplomacy.

The patient should be placed in a room with a constant temperature in which the atmosphere is freely renewed, yet without draught. The best way is to have two rooms for the patient, in one of which is placed the bed while in the other one window is always open. If only one room is available, the patient's bed should be placed opposite the window and he should be isolated by means of a screen. In winter, fire, preferably of wood, will maintain the temperature constant as required. Briefly, a light room, well ventilated, with a constant temperature, should be the dwelling of a scarlatina patient. Some physicians like to spray antiseptics in the air of the room, the therapeutic value of which is questionable but the moral value of which upon the patient and his surroundings is not to be disdained. A solution of carbolic acid, five parts in a thousand, may be sprayed in the room or else some water to which a teaspoonful of eucalyptol is added. Still more simple is the method of evaporating slowly in a dish a glass full of water in which are placed twenty grammes of each of eucalyptol and tincture of benzoin together with two decigrammes of menthol.

Red light, which has been used especially in smallpox in which it is said to lessen the severity of the eruption, has also been used in scarlatina, as for example by Schoull and Chop. These authors find that the eruption is very much less marked and the desquamation is very much milder, the fever is lower and of shorter duration, in short, that the disease is more benign and is shorter when the patient is exposed to red light. Although their cases are still insufficient to allow of a definite conclusion, the use of red light is harmless and deserves a trial. Red paper may be pasted on the glass panes of the room, thus procuring the desired color.

*Local and General Antiseptics.*—These methods are intended to disinfect the skin and the natural orifices of the body, thus preventing in a large measure the complications of the disease. *Antisepsis of the skin.* The purpose of this is to reduce the itching or burning sensation which accompanies the eruption. For this purpose we alternate enunctions of fats with warm baths Every other day the patient should be anointed from head to foot with a flannel soaked in olive oil or in fresh lard. On the intervening days the patient will take a lukewarm bath in which a handful of borax has been dissolved. Warm baths have a rather bad reputation with the laity in the treatment of eruptive fever, yet we may succeed in securing them if we insist that they are useful in making the eruption "come out." If they are refused we must content ourselves with warm lotions. But these should be absolutely insisted upon.

Asepsis of the mucous membrane. A variety of ways are used for the disinfection of the nose. Karslaw uses bougies of iodoform and eucalyptol. Nasal douches tend to excite an otitis. The best way to do is to insufflate into each nostril twice a day some powdered boric acid or else to introduce a few drops of the following preparation:

℞ Boric acid .................................... 2 grammes
Glycerine ....................................10 grammes
or, Mentholated vaseline, ................... 5 grammes

The mouth and throat should be kept aseptic with large irrigations of boric acid three times daily. In the intervals the patient should gargle freely with dilute peroxide of hydrogen and his throat should be painted with the following solution applied upon an applicator with cotton:

| | |
|---|---|
| ℞ Salicylic acid | 1 gramme |
| Alcohol | 2 grammes |
| Glycerine | 18 grammes |

The intestinal mucosa should also be watched especially in patients whom milk constipates. Mild purgatives such as castor oil, sodium sulphate and calomel in children (avoiding mercurial poisoning of course) will assure intestinal asepsis. The genital mucous membranes, especially in little girls and in hospitals, will demand daily care. They should be washed every morning with Van Swieten's solution diluted with five times its volume of boiled water.

*The Diet of Scarlet Fever Patients.*—This is one of the disputed points in the treatment of scarlet fever and one of the most embarrassing sometimes to the physician. The laity has heard something of the recent discussions on the comparative value of milk diet and chlorine-free diet and of the use of these diets in cases of nephritis. In cases without albumen in the urine the physician might alarm the parents by prescribing one of these diets; then there is the question when he should prescribe one of these diets, and when the other. Opinions vary on this subject, as we shall see from the following brief sketch:

A few years ago every patient with scarlet fever who had albumen in the urine was placed upon a milk diet for a more or less prolonged time. Guinon prohibits alcoholic drinks, puts the patients to bed and gives them acid drinks and milk, feeding them also with soups after the fever has subsided. Hutinel is still more strict and prescribes absolute milk diet for four or five weeks. Legendre and Broca are not so strict and on the fifteenth or twentieth day modify the milk diet by the addition of soups and eggs.

But soon a diametrically opposed view began to gain ground. In 1905 Dufour reported 200 cases in which he had fed the patient with soups, bread, meat and obtained no unfavorable results, but, on the contrary, improved the patient's strength and made him capable of resisting the complications of the disease. Beclare, Sireduy and Comby endorsed this opinion with some reservation and modified this diet only when there were traces of albumen in the urine.

Finally, Dopter concluded that a chlorine-free diet, which is useful in nephritis, would prevent the latter in scarlet fever. He therefore gave the patient meat without salt and his views were later confirmed by Guinon and Pater. The following diet is a compromise of these various views and is suitable for the average case of a non-albuminuric scarlet fever:

(*a*) During the first four or five days when the patient is under the full sway of the disease and has no desire for solid food a milk diet should be maintained. From one-half to two litres should be given daily, according to age, and in the intervals the patient may have sour drinks such as lemonades, grape juice, etc.

(*b*) After the fifth or sixth day we retain the milk as a beverage and add to it soups, eggs, without salt, and, later, vegetable pastes, purees and meat without salt. This salt-free diet should be continued until the end of the third week of the disease, when we should begin to add salt gradually until the end of the sixth week, when the patient can eat what he likes.

Pediatrists have not yet adopted the above diet and most of them still recommend milk diet during the entire disease.

*Prophylactic Measures.*—We shall not dwell upon the commonplace rules known to every physician concerning the wearing of gowns which shall remain in the room and should be disinfected; the careful washing of hands and their disinfection; the fumigation of the premises after the recovery. It is the duty of the physician to supervise the application of antiseptic principles on the part of the attendants, who are more skeptical and less educated than he. But in addition two problems present themselves to the practitioner owing to the divergence of opinion concerning them.

The first is, how long shall we isolate the patient? Authors differ in this respect, some giving thirty and some fifty days for a scarlet fever without complications. If the patient has been treated with warm baths alternating with inunctions of fat, as we have described, the period of desquamation will be so short that thirty days will be sufficient. In order to be quite sure that all danger of contagion is past, let us say forty days. We must remember, however, that any patient with scarlatina who has a suppurative complication should be isolated until the focus of suppuration is no longer active. Cases have been reported in which contact with scarlatina patients having suppurative foci at the sixtieth day was followed by contagion.

The second problem is, shall we stop nursing scarlatinal infants? There are two ways of looking at this problem. In the first class of cases the mother or nurse has scarlet fever. Some authors recommend weaning in such cases and say that there are exceptional cases of scarlet fever in infants. Dufour is correct, however, in saying that the danger of artificial feeding for an infant is greater than the remote possibility that he may be infected. In view of the singular immunity of infants against scarlet fever, therefore, the infected nurse or mother should continue to feed the child. In other cases the infant has the disease and then either the nurse has had scarlet fever and may continue to nurse him, or she has not had it and should then interrupt nursing. Whenever nursing is continued strict isolation of nurse and child should be enforced during the entire period of exclusion imposed by the disease.

